# Guided Internet-Based Cognitive Behavioral Therapy for Insomnia: Health-Economic Evaluation From the Societal and Public Health Care Perspective Alongside a Randomized Controlled Trial

**DOI:** 10.2196/25609

**Published:** 2021-05-24

**Authors:** Claudia Buntrock, Dirk Lehr, Filip Smit, Hanne Horvath, Matthias Berking, Kai Spiegelhalder, Heleen Riper, David Daniel Ebert

**Affiliations:** 1 Chair of Clinical Psychology and Psychotherapy Friedrich-Alexander University Erlangen-Nuremberg Erlangen Germany; 2 Institute of Psychology Leuphana University Lueneburg Lueneburg Germany; 3 Center for Economic Evaluation and Machine Learning Trimbos Insitute Utrecht Netherlands; 4 Department of Biostatistics and Epidemiology Amsterdam Public Health Research Institute VU University Medical Center Amsterdam Amsterdam Netherlands; 5 Department of Clinical, Neuro and Developmental Psychology Vrije Universiteit Amsterdam Amsterdam Netherlands; 6 GET.ON Institute Hamburg Germany; 7 Department of Psychiatry and Psychotherapy Medical Center University of Freiburg Freiburg Germany; 8 Telepsychiatric Centre University of Southern Denmark Odense Denmark; 9 Department of Sport and Health Sciences Technical University of Munich Munich Germany

**Keywords:** insomnia, internet-based cognitive behavioural therapy, iCBT-I, economic evaluation, cost-effectiveness, cost-utility, cognitive behavioral therapy, behavior, sleep, economics, public health, perspective

## Abstract

**Background:**

The evidence base for internet-based cognitive behavioral therapy for insomnia (iCBT-I) is firm; however, little is known about iCBT-I’s health-economic effects.

**Objective:**

This study aimed to evaluate the cost-effectiveness and cost–utility of iCBT-I in reducing insomnia among schoolteachers.

**Methods:**

Schoolteachers (N=128) with clinically significant insomnia symptoms and work-related rumination were randomized to guided iCBT-I or a wait list control group, both with unrestricted access to treatment as usual. Health care use, patient and family expenditures, and productivity losses were self-assessed and used for costing from a societal and a public health care perspective. Costs were related to symptom-free status (score <8 on the insomnia severity index) and quality-adjusted life years (QALYs) gained. Sampling error was handled using nonparametric bootstrapping.

**Results:**

Statistically significant differences favoring the intervention group were found for both health outcomes (symptom-free status yes or no: β=.30; 95% CI 0.16-0.43; QALYs: β=.019, 95% CI 0.01-0.03). From a societal perspective, iCBT-I had a 94% probability of dominating the wait list control for both health outcomes. From a public health care perspective, iCBT-I was more effective but also more expensive than the wait list control, resulting in an incremental cost-effectiveness ratio of €650 per symptom-free individual. In terms of QALYs, the incremental cost-effectiveness ratio was €11,285. At a willingness-to-pay threshold of €20,000 per QALY gained, the intervention’s probability of being cost-effective was 89%.

**Conclusions:**

Our trial indicates that iCBT could be considered as a good value-for-money intervention for insomnia.

**Trial Registration:**

German Clinical Trial Registry: DRKS00004700; https://tinyurl.com/2nnk57jm

**International Registered Report Identifier (IRRID):**

RR2-10.1186/1745-6215-14-169

## Introduction

Insomnia is characterized by difficulties in initiating or maintaining sleep and/or early morning awakenings that occur 3 nights or more per week, for at least 3 months, resulting in poor sleep quality and significant daytime impairment [1]. Insomnia is one of the most common sleep disorders among adults. Prevalence estimates range from 6% for insomnia disorder [2] to 25% for insomnia symptoms [3].

Insomnia is associated with a range of adverse health consequences for individuals, including poor daytime functioning [4] and reduced health-related quality of life [5]. In view of its high prevalence and its debilitating nature, insomnia is related to a substantial health and economic burden. As such, it increases the risk of future mental disorders (eg, major depressive disorder) [6]. Economic costs stem from both absenteeism and reduced productivity while at work (ie, presenteeism) [7] as well as increased health care utilization, (eg, medication prescription [8,9]).

In order to reduce the personal and economic burden of insomnia, it is essential to implement interventions that can improve sleep. American and European guidelines recommend cognitive behavioral therapy (CBT) as first-line treatment for insomnia disorder [10,11] due to its substantial clinical evidence base [12]. However, despite this recommendation, CBT for insomnia (CBT-I) is not widely available, mainly due to a shortage of therapists and available resources [13]. In addition, only 37% of those suffering from insomnia seek professional help [3].

Internet-based CBT intervention for insomnia (iCBT-I) has been touted as a solution that can bridge this gap in health care [14]. Meta-analytic evidence demonstrated that iCBT-I is effective in treating insomnia with large effect sizes at posttreatment (eg, Cohen’s d = 1.09; 95% CI 0.74-1.45) for insomnia severity [15]. Effect sizes are comparable to those found in individual face-to-face delivered CBT-I (eg, d=1.11; 95% CI 0.94-1.28) [12].

Although the effectiveness of (i)CBT-I has been demonstrated, research on its economic costs and benefits is still limited [16]. Previous reviews have argued that treating insomnia costs less compared to doing nothing [17,18]. A recent review on CBT-I interventions (N=7) using a dominance ranking framework showed that CBT-I was cost-effective compared to pharmacotherapy or no treatment [19]. However, only 2 studies have evaluated the economic effects of an iCBT-I intervention, but they suggested that iCBT-I provides superior health improvements at reduced costs [20,21]. To the best of our knowledge, no study has yet investigated the economic merits of iCBT-I from a societal perspective (including reductions in direct medical costs, patient and family costs, and indirect costs stemming from productivity losses) and from the public health care perspective (including only direct medical costs).

The aim of the this paper was thus to assess, from a societal and public health care perspective, the cost-effectiveness and cost–utility of a guided iCBT-I intervention to reduce insomnia symptoms in currently employed schoolteachers. The health economic evaluation presented here was conducted alongside a randomized controlled trial [22]. A previous publication reported the clinical effects of the iCBT-I intervention (6-month follow-up: Cohen d=1.43; 95% CI 1.04-1.82) [23].

## Methods

### Study Design

We conducted and reported the health-economic evaluation in agreement with the Consolidated Health Economic Evaluation Reporting Standards (CHEERS) statement [24] and the guidelines from the International Society for Pharmacoeconomics and Outcomes Research (ISPOR) [25]. The economic evaluation was performed from a societal perspective (ie, all relevant costs) and a public health care perspective (ie, direct medical costs) alongside a pragmatic 2-armed randomized controlled trial to establish the cost-effectiveness and cost–utility of a guided iCBT-I intervention as an adjunct to treatment as usual (TAU) for schoolteachers with insomnia compared to a wait list control condition with unrestricted access to TAU. Self-report questionnaires to assess costs and effects were collected at baseline, posttreatment (only health effects; 8 weeks after randomization), and 6-month follow-up via a secured online-based assessment system (Advanced Encryption Standard, 256-bit encrypted). Full details of the trial design can be found elsewhere [22]. The study was approved by the ethics committee of the University of Marburg (reference number: 2013-01K) and is registered under DRKS00004700 in the German clinical trial registry.

### Procedure

Participants were recruited in Germany from March 2013 to September 2013 using email distribution lists to primary, secondary, and vocational schools, which were provided by the Ministry of Education in the German state of Nordrhein–Westfalen. Currently employed schoolteachers aged 18 and above with clinically significant insomnia symptoms (Insomnia Severity Index [ISI] >14) and elevated work-related rumination (Irritation scale, subscale “Cognitive Irritation” >14) were included in the study [22]. The exclusion criterion was current psychotherapy for insomnia and/or suicidal ideation (Beck Depression Inventory item on suicidality >1). People taking sleep medication were not excluded from the study but were required to keep their medication on a stable dose during the study period. The Consolidated Standards of Reporting Trials (CONSORT) study flowchart and participants’ characteristics at baseline can be found elsewhere [23]. In brief, 128 schoolteachers were recruited into the trial with 64 randomized to the intervention and 64 to the wait list control condition. The average participant was female (95/128, 74%), 48 years of age (SD 10), married or in a partnership (92/128, 72%), and had a diagnosis of a primary insomnia (100/128, 78.1%) with moderate severity, and 14% (18/128) had a comorbid major depression [23]. Randomization took place at the individual level in a ratio of 1:1 and was conducted centrally by an independent research staff member not otherwise involved in the study using an automated web-based program (randomisation.eu). Study participants were not masked to their treatment allocation due to the nature of the psychological intervention.

### Interventions

In this pragmatic trial, all study participants had unrestricted access to TAU. In Germany, TAU for elevated insomnia symptoms usually includes visits to the general practitioner followed by more intensive interventions, such as cognitive behavioral therapy and medication if insomnia symptoms persist or worsen.

#### iCBT-I Intervention (GET.ON Recovery)

The iCBT-I intervention (GET.ON Recovery [22,23]) has been specifically tailored to schoolteachers experiencing work-related stress and insomnia. The intervention was mainly based on cognitive behavioral methods (eg, sleep restriction therapy, stimulus control therapy, relaxation, sleep hygiene, and cognitive interventions) [26]. These methods were supplemented by techniques effective in reducing work stress and fostering mental detachment from work-related problems derived from behavioral activation [27], metacognitive therapy [28], gratitude research [29], and research on boundary management [30]. The intervention consisted of 6 weekly modules. Overall, of the 64 participants, 61 (95.3%) completed all 6 modules [23]. Participants received written feedback on each completed module by an eCoach (ie, a trained clinical psychologist), who followed a standardized coaching manual. To maximize the comparability of the participants and maintain the guidance at a minimal level, eCoaches were advised that the time spent on each participant per module should not exceed 30 minutes; thus, the total amount of time spent on each participant was approximately 3 hours for the total duration of the intervention [23]. eCoaches were supervised by a clinical psychologist.

#### Wait List Control Condition

In addition to TAU, individuals in the control group were eventually granted access to the unguided version of the intervention after completing the final assessment at 6 months post baseline.

### Outcomes

#### ISI Symptom-Free Status

The health outcome in the cost-effectiveness analysis was symptom-free status defined as a score <8 on the ISI [31]. The ISI is a 7-item instrument answered on a 5-point Likert scale with a total score ranging from 0 to 28. The psychometric properties of the online version of the questionnaire have been well established [32]. In the current study, internal consistency was set at Cronbach α=.91.

#### Quality-Adjusted Life Years

Quality-adjusted life years (QALYs) were used as a health outcome in the cost–utility analysis (CUA). QALYs were based on the 6D Health State Short Form (SF-6D; a subset of 6 items of the Short Form Health Survey Version 1 [33]). The SF-6D contains 6 dimensions (each with between 2 and 5 levels) and can generate 7500 different health states. Utility values were derived using Brazier’s algorithm [34]. QALY health gains were estimated by calculating the area under the curve of linearly interpolated SF-6D utilities between measurements to cover the whole 6-month follow-up period. The SF-6D was used in this study because the instrument is known to be more sensitive to changes in mild to moderate physical and mental health conditions than is the EQ-5D-3L questionnaire [35,36].

### Resource Use and Costing

We used the Trimbos and Institute for Medical Technology Assessment "Treatment Inventory of Costs in Patients with psychiatric disorders" questionnaire (TiC-P) to collect data on health care utilization, patient and family costs, and productivity losses [37,38]. The TiC-P is a retrospective questionnaire with a 3-month recall period. The TiC-P was adapted for use in Germany and has been used in a large number of cost-effectiveness studies [39-41]. Costs were expressed in euros and indexed for the year 2013, the year the study was conducted, based on the German consumer price index (index factor 1.04) [42]. A reference to the National Institute for Health and Care Excellence willingness-to-pay (WTP) threshold of £20,000 (€23,529) to £30 000 (€35,294) per QALY gained was made where appropriate [43]. Costs were converted to pound sterling (£) using the purchasing power parities reported by the Organization for Economic Cooperation and Development. For the reference year 2013, €1 was equated to £0.85.

#### Intervention Costs

At the time of conducting the study, the market price of the iCBT-I provided by the GET.ON Institute was €299 (£254) per participant including all costs for developing (eg, tailoring intervention content to the target group) and delivering the intervention (eg, eCoaches providing individual feedback to participants).

#### Health Care Costs

We used 2 German guidelines for calculating health care costs [44,45]. A list of unit cost prices (ie, outpatient care) was used to compute the total health care costs on a per-participant level. Unit cost prices indexed for the year 2013 were as follows: €20.92 (£17.78) for a visit to the general practitioner, €68.06 (£57.85) for an internal medicine consult, €46.55 (£39.57) for a session with a psychiatrist, and €81.44 (£69.22) for a session with a psychotherapist. Hospital stays were computed at €335.52 (£285.19) for an in-patient day in a psychiatric hospital and €306.41 (£260.45) for an in-patient day in a hospital for psychosomatic medicine and psychotherapy. Costs were estimated by multiplying the units of resource use with corresponding unit cost prices.

#### Medication

The costs of prescribed medication were based on the German drug registry (Rote Liste [46]). Costs of prescribed medication are calculated as the pharmacy retail price, with the pharmacist’s “clawback” (ie, wholesale margin) being accounted for. The rates of discount vary between private and statutory (public) health insurances. Therefore, we weighted the mean costs of the 3 largest packages with the same agent based on the daily defined dose by the statutory population share (89% of the German population are statutorily insured).

#### Patient and Family Costs

Out-of-pocket expenses were directly obtained from participants. Costs for traveling were valued at €0.30 (£0.26) per kilometer for making trips to access health services. Time spent on the intervention and costs of informal care were valued using the opportunity cost method and were estimated at €23.10 (£19.64) per hour [44,45].

#### Costs of Productivity Losses

Productivity losses can be caused by absenteeism (ie, days not worked) and presenteeism (ie, reduced efficiency while at work). We followed the human capital approach to value costs due to absenteeism [47]. Lost workdays due to absenteeism were valued at the corresponding gross average of participants’ income per day. Lost workdays due to presenteeism were computed by taking into account the number of work days for which the participant reported reduced functioning weighted by the reported corresponding inefficiency score for those days (Osterhaus method) [48]. Productivity losses from unpaid work (ie, household work) were valued using the replacement cost method [49]. The estimated value was €18.33 (£15.58) per hour (eg, the average hourly gross wage of domestic help).

### Analysis

The study was not powered to statistically test differences in health-economic outcomes. Therefore, we took a probabilistic decision-making approach to make health-economic inferences [50]. We did not discount costs and effects because the analysis was limited to a 6-month time horizon.

In evaluating clinical and cost outcomes, we reported all analyses in accordance with the CONSORT statement [51]. Data were analyzed according to the intention-to-treat principle. Missing data in ISI were imputed via multiple imputation using a Markov Chain Monte Carlo multivariate imputation algorithm (SPSS 21, IBM Corp) with 10 estimations per missing value [23]. To account for missing data in the cost and utility data, we used the regression imputation procedure in Stata version 16 (StataCorp) to obtain the required predicted values [52]. Predictors of outcome and dropout were identified by (logistic) regression analyses. Identifying predictors of outcome helped us to obtain the most likely values of the outcome, whereas identifying predictors of dropout allowed us to correct for bias that might arise by differential loss to follow-up. Differences in effectiveness between study groups at 6-month follow-up were estimated using ordinary least square regression analyses. Due to baseline imbalances, QALYs were adjusted for baseline values [53]. To compare cumulative costs between study groups, a generalized linear regression model with a gamma family distribution and an identity link function was fitted and adjusted for baseline depressive symptom severity and age. The family distribution was selected based on the modified Park test [54]. The identity link function was chosen because an additive effect of the covariates was expected.

Societal costs included all cost categories, while direct medical costs used in analyses from the public health care perspective comprised intervention costs, health care costs, and medication costs. In the cost-effectiveness analysis, the incremental cost-effectiveness ratio (ICER) was calculated by dividing incremental costs (total per-participant costs or direct medical costs) by symptom-free status gained. In the cost–utility analysis, incremental costs were divided by QALYs gained. The corresponding equation was as follows: ICER = (CostsINT – CostsCTR) / (EffectsINT – EffectsCTR), where INT is the intervention group and CTR is the control group [47]. Although costs were gamma distributed, the difference of 2 nonnormally distributed variables (eg, incremental costs) followed a remarkably normal distribution. Hence, to handle sampling uncertainty, we bootstrapped the seemingly unrelated regression equations model (“sureg” command in Stata) to generate 2500 simulations of incremental cost and incremental effect pairs while allowing for correlated residuals of the cost and effect equations and adjusting for potential confounders (eg, baseline utilities in the effect equation; age and baseline depressive symptom severity in the cost equation) [55]. Based on the bootstrapped seemingly unrelated regression equation model, bias-corrected and accelerated 95% CIs were obtained for incremental costs and effects. In addition, 95% CIs around ICERs were obtained by the bootstrap acceptability method [56]. The bootstrapped cost and effect pairs were graphically represented on a cost-effectiveness plane with effects along the horizontal axis and costs along the vertical axis [47]. To assess the probability of the intervention being cost-effective at varying WTP thresholds, cost-effectiveness acceptability curves were plotted [57]. Except for the imputation of missing ISI scores, all analyses were performed using Stata version 16 (StataCorp) [52].

### Sensitivity Analyses

Three sensitivity analyses were performed. Presenteeism has been previously identified as one of the main cost drivers in insomnia [20]. As there is no gold standard to measure costs due to presenteeism, we used a different approach to assess presenteeism costs in the first sensitivity analysis. Here, we calculated costs due to presenteeism based on the Health and Labor Questionnaire (HLQ) method. The Osterhaus method, used in the main analyses, tends to overestimate costs due to presenteeism because this method concentrates on the work capacity of the individual. In contrast, the HLQ method focuses on production loss that is recoverable and is not yet made up for, thus generating a lower estimate of costs [58]. In a second sensitivity analysis, we varied the costs of the intervention by plus and minus 20% and 50%, respectively, to reflect uncertainties about the actual market price also including a lower price due to scaling effects. Finally, we conducted a “completers-only analysis” based on the data of the participants who completed the 6-month follow-up assessment.

## Results

### Study Dropout

At posttreatment, 92.2% (118/128) of the participants completed the follow-up questionnaires, whereas at 6-month follow-up, 88.3% (113/128) did. Dropout rates did not differ between the intervention and control conditions (χ2_1_=1.89; *P*=.17). Study dropout was neither associated with baseline insomnia severity nor with any sociodemographic factors (lowest *P* value=.18 for treatment allocation). There were no missing data due to item nonresponse.

### Effects

In the intervention group, 27 out of 64 participants (42%) reached a symptom-free status, whereas in the control group, 4 out of 64 participants (6%) were symptom-free at 6-month follow-up [23]. Statistically significant differences favoring the intervention group were found between the intervention and control group in symptom-free status (incremental effect, (Δ[E]=0.30; 95% CI 0.16-0.43). On average, participants in the intervention group gained 0.36 QALYs (95% CI 0.35-0.37) during the study period, while participants in the control condition gained 0.35 QALYs (95% CI 0.34-0.36). Differences in adjusted incremental QALYs were statistically significant (Δ[E]=0.02; 95% CI 0.01-0.03).

### Costs

Both groups showed similar total costs within the 3-month recall period before randomization (intervention group: €2902 [£2467], 95% CI €2111-€3693; control group: €3112 [£2645], 95% CI €2321-€3903). Table 1 presents the 6-month accumulated per-participant costs separately for different cost categories by treatment allocation. Mean direct medical costs were higher in the intervention group (€592 [£503]; 95% CI €444-€740) compared to the control condition (€389 [£331]; 95% CI €242-€537), which could largely be explained by the intervention costs that were only involved in the iCBT-I group. In contrast, both patient and family’s costs, along with productivity costs, were lower in the intervention group compared to the control group, with costs related to productivity losses having the largest impact on overall societal costs. Mean per-participant total costs accrued over the 6-month follow-up period were €4030 (£3426; 95% CI €3125-€4934) for the intervention group and €5021 (£4268; 95% CI €3394-€6147) for the control condition. Adjusted incremental differences in total costs were in favor of the intervention group (Δ[C]=–€895 [–£761]; 95% CI –€2155 to €364; ie, lower by €895 in the intervention group).

**Table 1:** Mean cumulative per-participant costs (in €) by condition over a 6-month follow-up period (based on intention-to-treat sample; N=128).

Costs		Intervention group (n=64), mean (95% CI)	Control group, (n=64), mean (95% CI)	Incremental costs, difference (95% CI)
**Direct medical costs**				
	Intervention costs	299^a^	—^b^	299^a^
	General practitioner	36 (21 to 51)	51 (36 to 66)	–15 (–36 to 6)
	Mental health care	150 (56 to 244)	163 (69 to 257)	–13 (–146 to 120)
	Antidepressants	2 (0 to 5)	5 (2 to 8)	–3 (–7 to 1)
	Allied health services^c^	105 (34 to 176)	170 (99 to 241)	–65 (–166 to 36)
**Patient and family costs**				
	Informal care	884 (226 to 1541)	1260 (602 to 1918)	–376 (–1306 to 554)
	Domestic help	310 (147 to 474)	(132 to 459)	15 (–217 to 246)
	Out-of-pocket expenses^d^	45(7 to 84)	73 (35 to 112)	–28 (-82 to 27) ^a^
	Travel	8 (3 to 13)	15 (10 to 20)	–7 (–14 to 1)
**Productivity costs**				
	Absenteeism	1005 (473 to 1537)	1104 (573 to 1636)	–99 (–851 to 653)
	Presenteeism	1185 (747 to 1623)	1883 (1446 to 2321)	–698
Total costs		4030 (2951 to 5108)	5021 (3942 to 6099)	–991 (–2519 to 534)

^a^As intervention costs are fixed, no 95% CI is applicable here.

^b^Not applicable.

^c^Including physiotherapist, massage, occupational therapist, etc.

^d^For example, allied health services without prescription.

### Economic Evaluation

#### Societal Perspective

**Figure 1:**
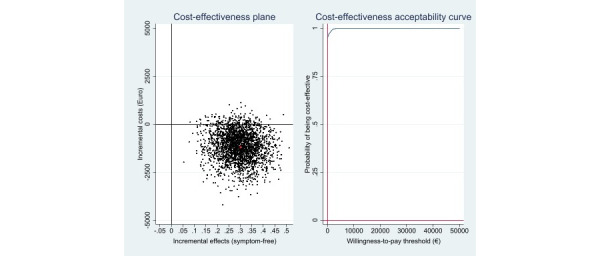
Scatterplot of 2500 replicates of the incremental cost and effect pairs (eg, symptom-free status) from the societal perspective on the cost-effectiveness plane: internet-based cognitive behavioral therapy versus wait list control condition and cost-effectiveness acceptability curve.

**Figure 2:**
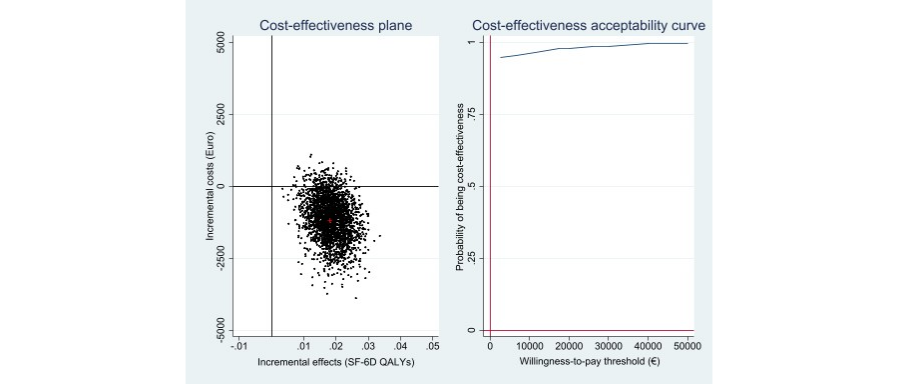
Scatterplot of 2500 replicates of the incremental cost and effect pairs (eg, QALYs gained) from the societal perspective on the cost-effectiveness plane: internet-based cognitive behavioral therapy for insomnia versus wait list control condition and cost-effectiveness acceptability curve. QALY: quality-adjusted life years; SF-6D: 6D Health State Short Form.

Table 2 shows the incremental cost, effects, and cost-effectiveness ratios (based on 2500 bootstrap simulations) for the main analyses. Cost-effectiveness analysis revealed that the iCBT-I intervention resulted in more symptom-free individuals (Δ[E]=0.30; 95% CI 0.16-0.43) and that these health gains were achieved at lower costs (Δ[C]=–€1121 [£953]; 95% CI –€3012 to €64). With regard to the cost-effectiveness plane, most of the replicated ICERs (94%) fell in the south–east quadrant, indicating a 94% probability that the intervention would dominate the control condition (Figure 1). Cost–utility analysis revealed similar results compared to the cost-effectiveness analysis (Table 2). Again, most of the bootstrapped cost and effect pairs (94%) fell in the south–east quadrant, indicating the dominance of the intervention over the control condition (Figure 2). When the societal WTP per additional QALY gained was €0, the iCBT-I intervention had a 94% probability of being more cost-effective than the control condition.

**Table 2:** Results of the main analyses (based on 2500 bootstrap simulations) based on societal and public health care perspectives.

Type of analysis			Incremental costs (in €), mean (95% CI)^a^		Incremental effects, mean (95% CI)^a^		ICER^b^, mean (95% CI)		Distribution over the cost-effectiveness plane (%)						
									NEQ^c^		NWQ^d^		SEQ^e^		SWQ^f^
**Societal perspective**															
	CEA^g^ (SFS)^h^	–1121 (–3012 to 64)		0.30 (0.16 to 0.43)		dominant^i^		6		—		94		—	
	CUA^j^ (SF-6D QALY)^k^	–1121 (–3012 to 64)		0.0183 (–0.0182 to 0.0185)		dominant		6		—		94		—	
**Public health care perspective**															
	CEA (SFS)	189 (–97 to 350)		0.30 (0.16 to 0.43)		650 (–215 to 1652)		94		—		6		—	
	CUA (SF-6D QALY)	189 (97 to 350)		0.0183 (0.0182 to 0.0185)		11,285 (–1750 to 27,493)		96		—		4		—	

^a^95% CIs in this column were bias-corrected and accelerated.

^b^ICER: incremental cost-effectiveness ratio.

^c^NEQ: north–east quadrant.

^d^NWQ: north–west quadrant.

^e^SEQ: south–east quadrant.

^f^SWQ: south–west quadrant.

^g^CEA: cost-effectiveness analysis.

^h^SFS: symptom-free status (0=no, 1=yes).

^i^dominant: The intervention resulted in higher effects at lower costs compared to the control condition.

^j^CUA: cost–utility analysis.

^k^SF-6D QALY: 6D Health State Short Form quality-adjusted life years based on the SF-12.

#### Public Health Care Perspective

**Figure 3:**
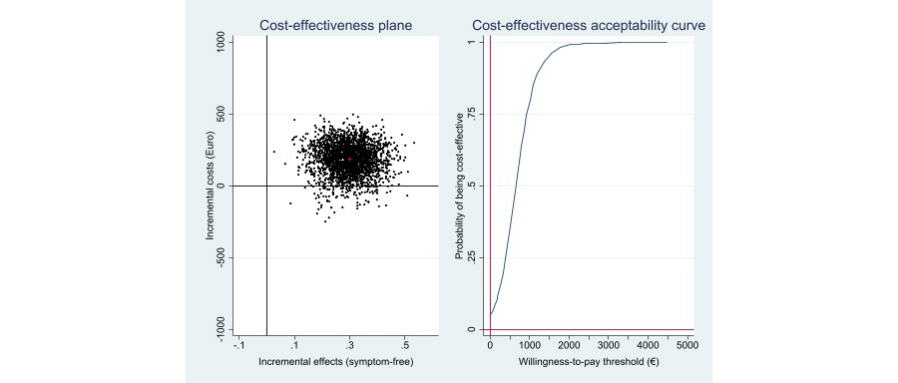
Scatterplot of 2500 replicates of the incremental cost and effect pairs (eg, symptom-free status) from the public health care perspective on the cost-effectiveness plane: internet-based cognitive behavioral therapy versus wait list control condition and cost-effectiveness acceptability curve.

**Figure 4:**
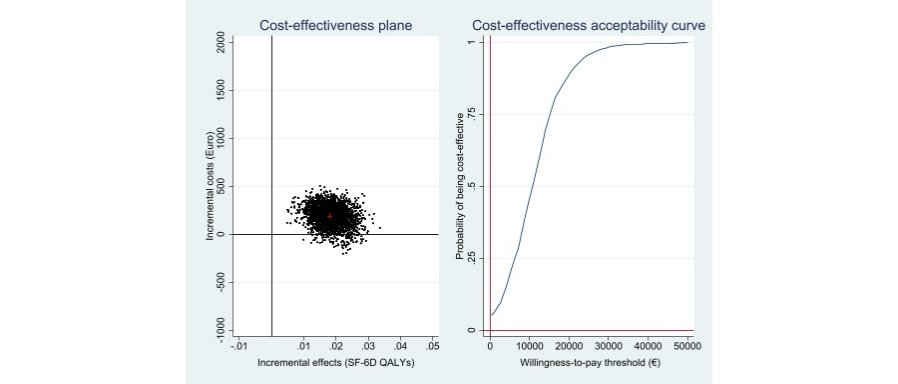
Scatterplot of 2500 replicates of the incremental cost and effect pairs (eg, QALYs gained) from the public health care perspective on the cost-effectiveness plane: internet-based cognitive behavioral therapy versus wait list control condition and cost-effectiveness acceptability curve. QALY: quality-adjusted life years; SF-6D: 6D Health State Short Form.

From a public health care perspective, health benefits were achieved at higher costs (€189 [£161]; 95%CI –€97 to €350). The ICER was €650 (£553; 95% CI –€215 to €1652) for 1 additional symptom-free individual. At a WTP threshold of €0, the iCBT-I intervention’s probability of being cost-effective was 6%. With an increase in the WTP to €1500 (£1275) per symptom-free status gained, the probability rose to 96% (Figure 3). Cost–utility analysis revealed an ICER of €11,285 (£9592; 95% CI –€1750 to €27,493) per QALY gained. The corresponding cost-effectiveness acceptability curve shows a probability of 4% and 89% that the intervention would be cost-effective at WTPs of €0 and €20,000 (£17,000) per QALY gained, respectively. With a National Institute for Health and Care Excellence WTP threshold of £20,000 (€23,529) to £30,000 (€35,294) per QALY gained [43], the probability would increase to 95% and 99%, respectively (Figure 4).

### Sensitivity Analyses

Cost-effectiveness estimates based on the completer-only sample were almost identical to the main analyses, indicating that the imputation procedure did not bias the results (Table 3). From a societal perspective, results of the sensitivity analyses showed that neither using the HLQ method to assess costs due to presenteeism nor increasing intervention costs affected the overall conclusion that the iCBT-I intervention produces greater health gains at lower costs compared with a wait list control condition (Table 3). Using the HLQ method, the intervention’s probability of being cost-effective was 72% at a WTP of €0 for both health outcomes. This probability rose to 86% at a WTP of €20,000 (£17,000) per QALY gained (Multimedia Appendix 1 Figure S1). Increasing or decreasing intervention costs by 20% or 50% did not affect the intervention’s probability of being cost-effective compared with the main analysis (Table 3). From a public health care perspective, with intervention costs decreased up to 50%, health effects were still gained at slightly higher costs. Increasing the intervention costs by 20% and 50% resulted in an ICER of €14,380 (£12,223) and €19,360 (£16,456) per QALY gained, respectively (Table 3). At a WTP of €20,000 per QALY gained, the intervention’s probability of being cost-effective was 80% and 60% (Multimedia Appendix 1 Figure S2), respectively.

**Table 3:** Results of the sensitivity analyses (based on 2500 bootstrap simulations) based on the societal and public health care perspective.

Type of analysis				Incremental costs (in €), mean (95% CI)^a^		Incremental effects, mean (95% CI)^a^		ICER^b^, mean (95% CI)		Distribution over the cost-effectiveness plane (%)						
										NEQ^c^		NWQ^d^		SEQ^e^		SWQ^f^
**Societal perspective**																
	**Presenteeism costs based on HLQ method^g^**															
		CEA^h^	–459 (–2155 to 796)		0.30 (0.16 to 0.43)		dominant^i^		28		—		72		—	
		CUA^j^	–459 (–2155 to 796)		0.0183 (0.0182 to 0.0185)		dominant		28		—		72		—	
	**Intervention costs plus 20%**															
		CEA	–1062 (–2801 to 192)		0.30 (0.16 to 0.43)		dominant		6		—		94		—	
		CUA	–1062 (–2801 to 192)		0.0183 (0.0182 to 0.0185)		dominant		7		—		93		—	
	**Intervention costs plus 50%**															
		CEA	–972 (–2804 to 249)		0.30 (0.16 to 0.43)		dominant		8		—		92		—	
		CUA	–972 (–2804 to 249)		0.0183 (.0182 to 0.0185)		dominant		9		—		91		—	
	**Intervention costs minus 20%**															
		CEA	–1181 (–3052 to 114)		0.30 (0.16 to 0.43)		dominant		5		—		95		—	
		CUA	–1181 (–3052 to 114)		0.0183 (.0182 to 0.0185)		dominant		5		—		95		—	
	**Intervention costs minus 50%**															
		CEA	–1271 (–3068 to 18)		0.30 (0.16 to 0.43)		dominant		3		—		97		—	
		CUA	–1271 (–3068 to 18)		0.0183 (.0182 to 0.0185)		dominant		3		—		97		—	
	**Completer analysis**															
		CEA	–1169 (–2963 to 625)		0.32 (0.18 to 0.46)		dominant		9		—		91		—	
		CUA	–1169 (–2963 to 625)		0.0188 (0.0089 to 0.028)		dominant		9		—		91		—	
**Public health care perspective**																
	**Intervention costs plus 20%**															
		CEA	246 (–59 to 405)		0.30 (0.16 to 0.43)		831 (59 – 1778)		98		—		2		—	
		CUA	246 (–59 to 405)		0.0183 (0.0182 to 0.0185)		14,380 (1135 to 31,826)		98		—		2		—	
	**Intervention costs plus 50%**															
		CEA	336 (38 to 503)		0.30 (0.16 to 0.43)		1129 (306 to 2137)		100		—		—		—	
		CUA	336 (38 to 503)		0.0183 (0.0182 to 0.0185)		19,360 (4671 to 40,673)		100		—		—		—	
	**Intervention costs minus 20%**															
		CEA	127 (–157 to 295)		0.30 (0.16 to 0.43)		413 (–400 to 1185)		87		—		13		—	
		CUA	127 (–157 to 295)		0.0183 (0.0182 to 0.0185)		7826 (–4120 to 22,565)		90		—		10		—	
	**Intervention costs minus 50%**															
		CEA	37 (–258 to 201)		0.30 (0.16 to 0.43)		129 (–636 to 838)		66		—		34		—	
		CUA	37 (–258 to 201)		0.0183 (0.0182 to 0.0185)		2384 (–9114 to 15,017)		66		—		34		—	
	**Completer analysis**															
		CEA	206 (–145 to 395)		0.32 (0.18 to 0.46)		656 (–296 to 1725)		92		—		8		—	
		CUA	206 (–145 to 395)		0.0188 (0.0089 to 0.028)		12,046 (–3920 to 33,174)		92		—		8		—	

^a^95% CIs in this column were bias-corrected and accelerated.

^b^ICER: incremental cost-effectiveness ratio.

^c^NEQ: north–east quadrant.

^d^NWQ: north–west quadrant.

^e^SEQ: south–east quadrant.

^f^SWQ: south–west quadrant.

^g^Costs due to presenteeism based on the Health and Labour Questionnaire method.

^h^CEA: cost-effectiveness analysis.

^i^dominant: The intervention resulted in higher effects at lower costs compared to the control.

^j^CUA: cost–utility analysis: quality-adjusted life years.

## Discussion

### Principal Results

Our study was set out to evaluate the cost-effectiveness and cost-utility of a guided iCBT-I intervention as an adjunct to usual care to reduce insomnia symptoms in schoolteachers in comparison with a wait list control condition with unrestricted access to TAU from a societal and a public health care perspective. Statistically significant differences favoring the intervention group were found for both health outcomes (symptom-free status and QALYs). From a societal perspective, the iCBT-I intervention dominated the wait list-control condition, meaning that the iCBT-I intervention has better health effects for less costs than does usual care in schoolteachers with insomnia. From a public health care perspective, the ICERs were €650 and €11,285 for a symptom-free individual and QALY gained, respectively. At a WTP threshold of €20,000 per QALY gained, the intervention’s probability of being cost-effective was 89%.

### Comparison With Prior Work

Although the effectiveness of iCBT-I is well established [15], there is a critical gap in health-economic evidence for iCBT-I. To our knowledge, this is the first trial-based economic evaluation of an iCBT-I intervention to reduce insomnia symptoms using a societal and public health care perspective. As such, results from our trial add to the converging evidence pointing to the cost-effectiveness of CBT interventions for insomnia. Thiart et al [20] used the same study to evaluate the cost and benefits of the iCBT-I intervention as seen from an employer’s perspective. Results of the current health-economic evaluation line up agreeably with these findings. Baka et al [21] compared a guided iCBT-I intervention to care as usual for insomnia patients in general practice. Analogous to our findings, mean societal costs were lower in the intervention group than in the care as usual group, and, in contrast to our results, the cost–utility analyses revealed a lower probability (69%) of the intervention being cost-effective compared to care as usual at a ceiling ratio of €30,000 per QALY gained. This difference could be due to different types of control conditions used (care as usual vs wait list control). Applying an employer’s or societal perspective seems to generate incremental costs favoring interventions groups when participants are employees, or at least in the productive age groups. In addition, our findings match with available health-economic evidence from a recent systematic review (N=7) showing that CBT-I was cost-effective compared to pharmacotherapy or no treatment [19].

Our findings from the public health care perspective showing that the iCBT intervention resulted in better health effects but achieved this at higher costs are also in line with findings from this systematic review [19]. Three trial-based economic evaluations employing a public health care perspective showed that CBT-I led to greater health improvements at higher costs compared to either TAU [59,60] or a wait list control condition [61], with time horizons ranging from 8 weeks to 6 months. In terms of QALYs gained, studies reported a low (34%) [61] and high (99%) [59] probability of CBT-I being cost-effective at a maximum WTP of £30,000 (€31,727) in the United Kingdom. Watanabe et al [60] reported a 90% chance of CBT-I being cost-effective at a WTP threshold of US$40,000 (€29,400) per QALY gained. In contrast, one study conducted from a public health care perspective showed that CBT-I was cheaper and more effective than TAU. However, there was large uncertainty around cost estimates resulting in a moderate probability (70%) of being cost-effective at a WTP of £30,000 (€31,727) [62].

### Limitations

This study has some limitations. First, the time horizon of this study was limited to 6 months. However, results of an economic model investigating the long-term cost-effectiveness of CBT-I among long-term hypnotic drug users with chronic sleep difficulties compared to TAU indicated that any increase in the timeframe of the economic evaluation produces substantial reductions in the incremental costs per QALY gained. The cost-effectiveness of CBT-I improved even when treatment effects were reduced radically over time [59]. Further studies should thus assess the long-term clinical and cost-effectiveness of iCBT-I to reaffirm its long-term cost-effectiveness. Second, the iCBT-I intervention was compared to a wait list control condition in the present trial. Although patients in the control group had full access to treatment as usual, we cannot rule out a potential nocebo effect in the wait list control condition [63]. In addition, pharmacoeconomic guidelines recommend standard care (eg, face-to-face CBT-I) as comparator [64]. Future studies should thus directly compare the cost-effectiveness of iCBT-I versus face-to-face CBT-I. Third, although the sample size in this trial was sufficient to demonstrate clinical effectiveness, it needs emphasizing that much larger sample sizes are required for hypothesis testing in economic studies due to the skewness of costs relative to normally distributed health effects [65]. Therefore, it is recommended that future studies employ larger sample sizes to allow for better evaluation of cost changes and sustainability of interventions like iCBT-I. Fourth, we only used the SF-6D to compute utilities and QALYs. However, the choice for the SF-6D (rather than its alternative the EQ-5D-3L [66]) matters, even to the point where decision-makers have to regard a new intervention as cost-effective or not [67]. In the current study, we flanked the cost–utility analysis (with QALYs) by a cost-effectiveness analysis (with improvements in insomnia) and both economic evaluations led to the same conclusion of the iCBT-I intervention being the preferred option. Nevertheless, future studies should employ different instruments to compute QALYs. Fifth, our results may only be generalized to professions with similar characteristics, such as flexible working hours, loose boundaries between work and private life, and work-home interference. Sixth, the trial has been conducted in a highly educated sample. Hence, we cannot predict the uptake of such an intervention in participants with a lower education level or among those with lower-income status. A recent individual participant data meta-analysis revealed, however, that education was not associated with differential treatment effects of an iCBT intervention to prevent depression [68] although other evidence suggests that better treatment adherence is predicted by higher education [69]. Attrition has been suggested to be an issue, especially in internet-delivered interventions [70]. It is thus warranted to conduct research into the willingness of specific population segments to fully engage in such interventions (ie, how uptake and adherence rates of iCBT interventions could be increased among individuals with less education). Finally, the research context might have led to a self-selection of individuals who might have been more motivated and committed to engage in the iCBT-I intervention than is assumed outside a research context. As a result, findings might not be generalizable to the wider population, but might be representative of specifically those willing to use internet-based interventions in the first place.

### Clinical Implications and Future Research

American and European guidelines recommend CBT as the first-line treatment for insomnia disorder [10,11]. Our study supports this recommendation by showing that a guided iCBT-I intervention may reduce insomnia symptoms and improve health-related quality of life. iCBT interventions for mental disorders have often been introduced as potential cost-saving alternatives to face-to-face individual or group therapy [71,72]. Findings from our study add to the evidence base that delivering cognitive-behavioral therapy over the internet has a high probability of being cost-effective in reducing insomnia symptoms among employees. In view of scarce resources and rising costs in health care systems, evidence-based guidance regarding cost-effectiveness of iCBT-I can potentially help to inform decision-makers to the choice of first-line treatments of insomnia. However, future studies should directly compare iCBT-I with face-to-face–delivered CBT-I.

Considering the shortage of therapists and available resources [13], a rather low uptake of professional help [3], and the potential to scale up iCBT-I interventions to efficiently alleviate the health and economic burden caused by insomnia, it would be worthwhile to integrate this type of intervention into routine practice. However, some risks need to be taken into account when scaling up this intervention. Involving individual eCoaches may hamper scaling up the intervention. The support offered by an eCoach may not only affect clinical effectiveness and cost-effectiveness of the intervention but also the target group’s willingness to participate in this intervention, thereby influencing intervention effects at the population level. Thus, future studies should compare the acceptability, effectiveness, and cost-effectiveness of guided and unguided iCBT-I interventions. In addition, there are no guarantees that adherence and (by proxy) effectiveness will be maintained if this sort of unsupported iCBT-I intervention is scaled up in the population. Finally, the same technical resources available in the research setting (eg, stable and secure internet connections) may not be available when the intervention is scaled up.

### Conclusions

Findings from our trial indicate that iCBT could be considered as good value for money in insomnia therapy. Given the evidence for the effectiveness of iCBT-I interventions to reduce insomnia symptoms, the potential scalability and cost-effectiveness of these interventions might strategically pave the way to alleviate the health and economic burden related to insomnia disorder (and its sequelae) in an affordable way. However, before a nationwide dissemination can be considered, future studies need to evaluate the comparative clinical and economic outcomes of guided and unguided iCBT-I interventions, and to determine what works best for whom such that the deployment of the intervention can optimally target the right population segments. Moreover, implementation studies are needed to clarify the real-world effects of these interventions and to gain insights into the willingness of specific population segments to fully engage in them (eg, individuals with a low education level or those of low-income status).

## Appendix

Multimedia Appendix 1 Additional figures.

Multimedia Appendix 2 CONSORT-EHEALTH Checklist (V.1.6.1).

## Abbreviations

(i)CBT-I: (internet-based) cognitive behavioral therapy for insomnia
CHEERS: Consolidated Health Economic Evaluation Reporting Standards
CONSORT: Consolidated Standards of Reporting Trials
HLQ: Health and Labor Questionnaire
ICER: incremental cost effect ratio
ISI: Insomnia Severity Index
ISPOR: International Society for Pharmacoeconomics and Outcomes Research
QALY: quality-adjusted life years
SF-6D: 6D Health State Short Form
SFS: symptom-free status
TAU: treatment-as-usual
TiC-P: Treatment Inventory of Costs in Patients with psychiatric disorders
WTP: willingness to pay
