# Supplementary material for: Guided Internet-Based Cognitive Behavioral Therapy for Insomnia: Health-Economic Evaluation From the Societal and Public Health Care Perspective Alongside a Randomized Controlled Trial
Source: J Med Internet Res. 2021 May 24;23(5):e25609. doi: 10.2196/25609 (PMC8185611; doi:10.2196/25609)

Multimedia Appendix 1

Figure S1. Cost-effectiveness acceptability curve showing the probability of iCBT-I being cost-effective at varying willingness-to-pay ceilings (based on 2,500 replicates of the incremental cost-effectiveness ratio using mean differences in costs from a societal perspective with costs due to presenteeism based on the HLQ method and quality-adjusted life years (QALYs)).

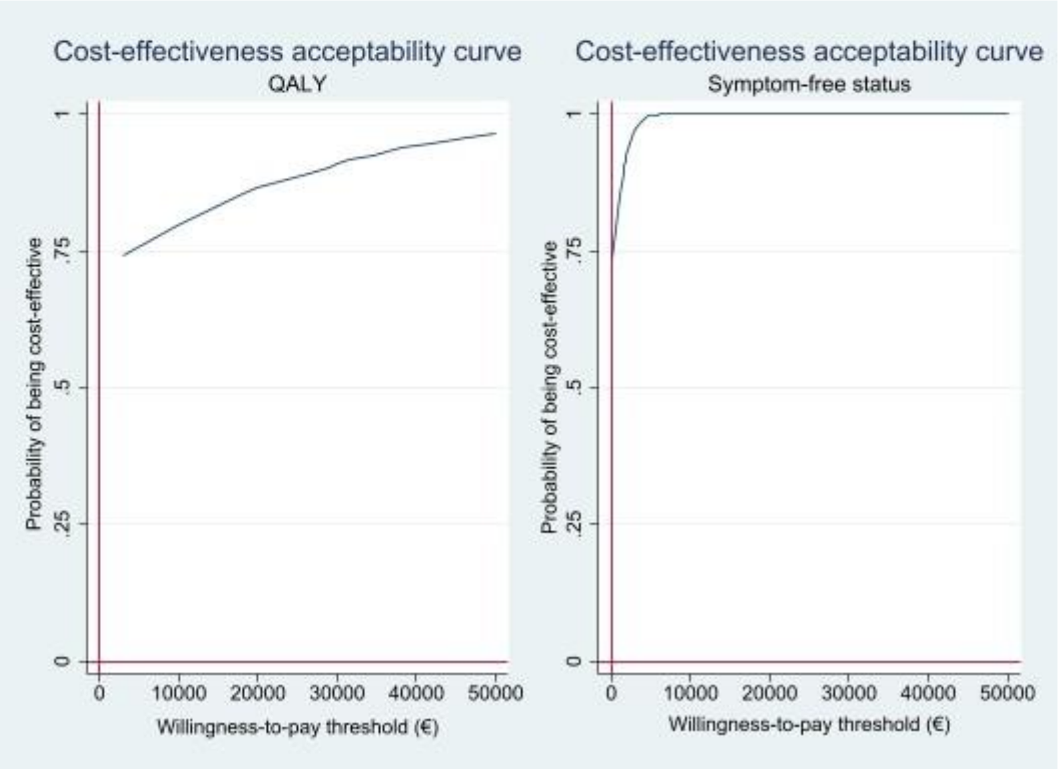

Figure S2. Cost-effectiveness acceptability curve showing the probability of iCBT-I being cost-effective at varying willingness-to-pay ceilings (based on 2,500 replicates of the incremental cost-effectiveness ratio using mean differences in costs from a societal perspective with intervention costs increased by 20% and 50%, respectively, and quality-adjusted life years (QALYs)).

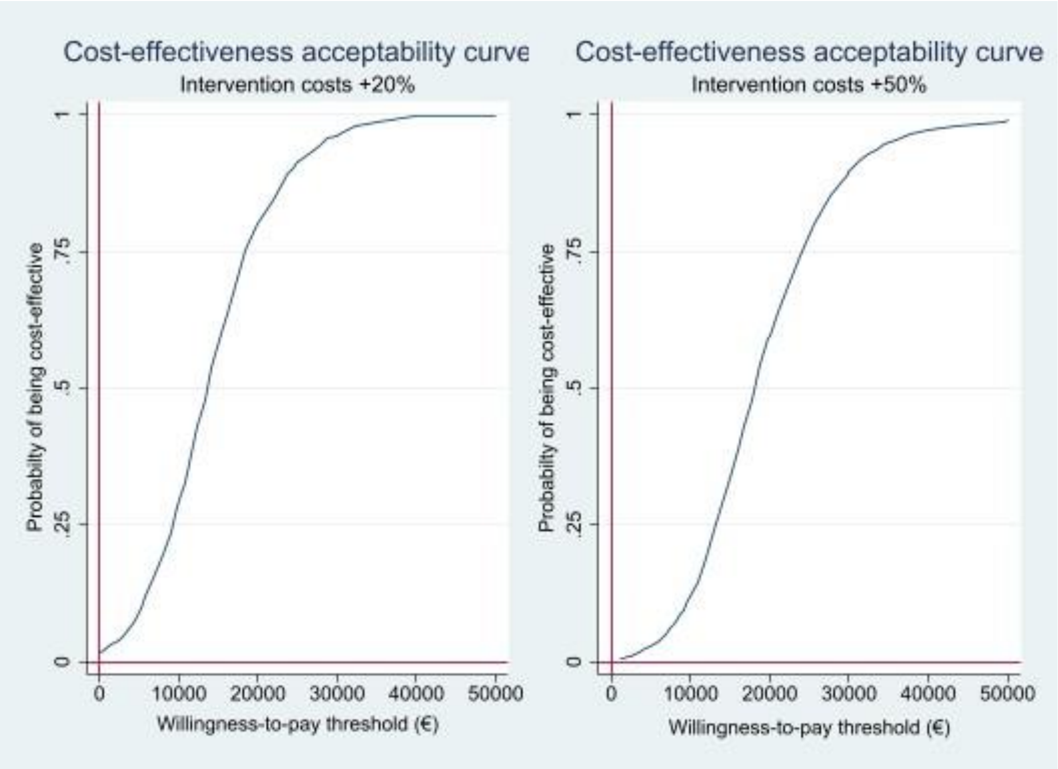

Supplement: Multimedia Appendix 1 [file jmir_v23i5e25609_app1.pdf]
